# Administration of Anæsthetics.—II

**Published:** 1895-11-30

**Authors:** Benjamin Ward Richardson


### Administration of Anæsthetics.—II.

By Sir Benjamin Ward Richardson, M.D., F.R.S.

### Constitution of an Anæsthetic.

Since the publication of my last paper, on November 2nd, the fatal accidents from chloroform in this country continue without abatement. They average, in this small section of Britain, more than one per week, and there may be more that are not recorded. Nitrous oxide has caused one death, ether three, mixtures four, and pure chloroform 47 in the same year, 1895, and these have each given origin to an inquest. How many more have occurred abroad and in neighbouring countries it is impossible to say, but they must have been numerous. It may be observed that chloroform is the chief cause of these deaths, and next the mixtures of which it forms a part; forty-seven being by chloroform, four by mixtures. It may be urged that as chloroform is so much more frequently used than the other anæsthetics, the cause of death lies in the numbers of administrations; but this can hardly be true, as, at the present time, ether is so often used instead of chloroform; and, certainly, forty-seven to four is a very unlikely estimate, based merely on numbers. Clearly, I think that the excessive frequency of death must rest either on the quality of the chloroform itself, on its mode of administration, or on some peculiarity of the persons to whom the anæsthetic has been administered.

It does not seem that it is always the difference between ether and chloroform that constitutes the danger. I notice, for instance, in the last case, where a woman of 33 died, that the cause of death was from ether. She had twice taken chloroform and awakened to a good recovery. She took ether once, and died in twenty minutes while the operation for reduction of a hernia was in progress. All this requires to be carefully remembered.

For our parts, we have to consider the question whether the fatalities from chloroform are due to that substance, or to the mode of its administration. I have dealt at length in previous papers upon mode of administration, and have suggested that death must be due to the large dose of chloroform inhaled. I am supported in that view by an experiment I made this very day on myself, and have been surprised to learn what a very small quantity of chloroform tends to produce sleepfulness, confusion, and the first stage of anæsthetisation. I inhaled from a flannel-covered cage held over the mouth, chloroform being administered in very minute quantities of vapour diffused into the cage by a gentle current of air driven through it. By the time I had inhaled nine minims, thus diffused, a part of which must have been repelled by the expiration, I certainly lost the complete stage of consciousness and entered into that of anæsthesia. If I had inhaled for double the length of time I should have certainly been quite unconscious, yet the quantity I had inhaled amounted only to nine minims, and gave no feeling whatever of irritation in the throat such as is felt when chloroform is directly inhaled without thought as to quantity. There must, therefore, be a great deal in inhalation, a fact I further proved by inhaling it in a more potent form, that caused some irritation in the throat, a little cough, a slight sense of nausea, and some rapidity of the circulation. Here, then, is a lesson that chloroform should be well diluted, and not administered in too concentrated a dose. If it be so, then other conditions follow; that is to say, there is more irritation of the throat, more frequent cough, more certain nausea, and there is greater quickening of the circulation. Forty years ago I was well acquainted with these facts, but I am acting correctly in repeating them. At the same time, both Snow and myself knew that eighteen minims of chloroform received into the blood were fully sufficient to induce a sufficiently deep unconsciousness for the action of the surgeon, and we must not, certainly, be surprised if on administration of very large doses to young children the result should be so unfavourable as it has been so often of late.

While these thoughts were originally on my mind I began to turn to the consideration of the constitution of an anæsthetic, and as to whether that might not lead to great difference in action. I conceived it more than probable that the effects of foreign vapour in the larynx might have a bad effect, and whether it was not worth while to study anæsthesia in connection with the constitution of the anæsthetic. It is pretty clear that nitrous oxide produces insensibility by the production of asphyxia, an idea which caused me at first to question the value of nitrous oxide as an anæsthetic, since it is certain that during its administration to inferior animals, and even to man, symptoms of asphyxia are developed before the signs of perfect anæsthesia were perfected. That is a fact which has been maintained up to the present hour, and the very last time I saw nitrous oxide given to a medical friend for the extraction of a tooth, I could not but observe that asphyxia was so strong a symptom that the patient looked precisely as if a cord had been tightened around his neck. I surmised also that ether acts very nearly in the same way, just as Faraday originally said it did; that is to say, like nitrous oxide, and I repeatedly witnessed the fact that under deep etherisation the etherised person breathed with the difficulty of asphyxia, and the administration had for the moment to be discontinued. These thoughts led me to make the inquiries on which so many of my early labours rested: inquiries detailed largely in my synopsis of anæsthetics, and consisting in taking one anæsthetic after another and determining upon its effects during inhalation.

No difficulties occurred to me in fixing attention at once on the chloride series, or, as they are sometimes called, the haloids. It was manifest that chlorine, however diluted, is a direct irritant to the larynx, the pharynx, the stomach, and the heart. It very easily killed by its irritable qualities, and one of my contemporaries, the late Dr. Scott, died actually in a bath in which chlorine was being liberated, and, as the late Dr. Markham, who was called to him, believed, he died directly from the action of the chlorine.

